# Surgical Versus Non-Surgical Management of Displaced Midshaft Clavicle Fractures: A Systematic Review and Meta-Analysis

**DOI:** 10.7759/cureus.93902

**Published:** 2025-10-05

**Authors:** Abdelfatah M Elsenosy, Eslam Hassan, Mustafa Al-Alawi, Ahmed S Yousef, Wael Elbagory, Senthil Muthian

**Affiliations:** 1 Trauma and Orthopedics, University Hospital Dorset, Poole, GBR; 2 Trauma and Orthopedics, Poole General Hospital, Poole, GBR; 3 Anesthesia, Southend University Hospital, Southend-on-Sea, GBR

**Keywords:** clavicle fracture, functional outcome, meta-analysis, midshaft, non-operative management, surgical fixation, union rate

## Abstract

Displaced midshaft clavicle fractures are common, particularly among young, active adults, and while both surgical and non-surgical approaches are used, their comparative effectiveness remains debated. This systematic review and meta-analysis evaluated surgical versus non-surgical management of acute displaced midshaft clavicle fractures in adults, focusing on union rates, functional outcomes, complications, and patient satisfaction. A comprehensive search of PubMed, Scopus, Web of Science, and Google Scholar was conducted up to May 2025, including studies involving adults with acute displaced midshaft fractures that reported at least one relevant clinical outcome. Meta-analyses were performed using RevMan 5.4 (The Cochrane Collaboration, London, England, UK), with standardized mean differences (SMDs) for continuous outcomes and odds ratios (ORs) for dichotomous outcomes. Eleven studies (1,084 patients) met inclusion criteria; surgical treatment significantly reduced nonunion rates (OR: 0.23; p < 0.00001) and showed modest improvements in early shoulder function (Constant score SMD: 0.49; p = 0.05), while DASH (Disabilities of the Arm, Shoulder and Hand) scores were similar (p = 0.35). Surgery also improved cosmetic satisfaction and shortened return-to-work time, but carried higher risks of hardware-related complications and reoperation. In summary, surgical fixation provides superior early outcomes and lower nonunion rates, especially in active patients, while long-term functional results are comparable between approaches, highlighting the importance of individualized treatment decisions based on fracture characteristics, patient activity level, and preferences.

## Introduction and background

Clavicle fractures are among the most common orthopedic injuries, accounting for approximately 2.6% to 5% of all adult fractures and up to 44-66% of shoulder girdle injuries. Midshaft fractures are the most prevalent subtype, representing 70-85% of all clavicle fractures [1,2], particularly affecting young, active individuals due to high-energy mechanisms such as sports injuries and road traffic accidents [3]. These fractures typically result from a direct blow to the shoulder or a fall onto an outstretched hand, transmitting axial force through the clavicle and causing failure at its structurally weakest midshaft point [4,5]. The clavicle’s subcutaneous location and narrow diaphyseal architecture further contribute to its susceptibility to injury.

While nondisplaced midshaft fractures often heal well with conservative treatment, displaced fractures are associated with higher rates of nonunion (up to 15%) and symptomatic malunion [6,7]. Complications may include chronic pain, cosmetic deformity, shoulder weakness, and impaired function-particularly concerning in physically active populations. Rarely, high-energy trauma may lead to neurovascular compromise, including brachial plexus irritation or musculocutaneous nerve injury [8].

Surgical management of displaced midshaft clavicle fractures generally yields high union rates and earlier return to function. For example, a prospective case series using the Anser Clavicle Pin reported 100% union at one year [9], and technical reports have demonstrated consistently high union with plate fixation, although healing times may vary with fracture complexity [10]. Functional outcomes following operative fixation are favorable, particularly in the early postoperative period. Studies report high Constant-Murley Scores (CMS), assessing shoulder function including pain, daily activities, range of motion, and strength, and low Disabilities of the Arm, Shoulder and Hand (DASH) scores, reflecting near-normal shoulder function [9,11]. Operative patients also typically return to work or activity sooner than nonoperative counterparts [12]. However, hardware-related complications such as implant irritation, deformation, or protrusion may occur and occasionally necessitate revision surgery [9].

Conservative treatment remains a valid option, particularly for less active individuals or fractures with minimal displacement. Although healing is generally satisfactory, nonunion and delayed union are more frequent. One comparative study reported a mean union time of 16 weeks in conservatively treated patients, with a higher incidence of nonunion compared to the surgical cohort [13]. Fracture displacement and comminution correlate with prolonged healing, whereas younger patients and simple fracture patterns show more favorable outcomes [14]. Patient satisfaction with nonoperative treatment is often influenced by cosmetic outcomes. A long-term study demonstrated that despite comparable shoulder function at five years, patients treated nonoperatively were less satisfied due to deformity or malalignment [15]. Vertical displacement exceeding 100% was strongly associated with patient preference for delayed surgical intervention due to cosmetic concerns [16].

Long-term functional outcomes are generally comparable between surgical and non-surgical groups. Several studies report similar CMS and DASH scores beyond one year, although operative groups frequently exhibit earlier functional recovery and better aesthetic satisfaction [15-18]. The treatment paradigm shifted in the early 2000s following pivotal randomized trials demonstrating lower nonunion rates, faster return to function, and improved cosmetic satisfaction with surgical fixation [7,19]. Recent comparative studies continue to support surgical management for early functional benefits and reduced nonunion or malunion rates [12,20]. Clavicle shortening and angulation have been implicated in poor biomechanical outcomes, including reduced shoulder strength, impaired range of motion, decreased endurance, and altered scapulothoracic kinematics, reinforcing the rationale for surgical correction in specific fracture patterns [20,21].

Multiple patient- and fracture-specific factors influence treatment decisions. Surgical fixation is more frequently advocated in younger, active individuals, particularly those with dominant limb involvement, high physical demands, or fractures with displacement >100% or shortening >2 cm, as suggested by Zhang et al. and other authors [22,23]. Comminuted fractures and superior-inferior displacement patterns are also predictors for surgical management, particularly in adolescents [22]. Socioeconomic and healthcare-related factors may further impact treatment selection, with patients having private insurance or treated in adult hospitals more likely to receive surgery [22]. While operative management offers superior early outcomes, long-term functional differences remain modest. Current evidence emphasizes individualized treatment strategies based on fracture characteristics, patient activity level, cosmetic expectations, and risk tolerance [24].

Despite extensive comparative research, uncertainty remains regarding which subgroups derive the greatest long-term benefit from surgery. Future studies should prioritize patient-centered decision-making and long-term outcome measures to optimize treatment strategies.

## Review

Methods

Search Strategy

A comprehensive systematic literature search was conducted using PubMed, Scopus, Web of Science, and Google Scholar to identify studies comparing operative and non-operative management of displaced midshaft clavicle fractures in adults. The search included literature published in the last 10 years, up to May 2025. Search terms and Medical Subject Headings (MeSH) were used in various combinations with Boolean operators and included: “clavicle fracture,” “midshaft clavicle,” “displaced clavicle,” “operative treatment,” “non-operative treatment,” “conservative management,” “ORIF,” “DASH score,” and “Constant score.” A comprehensive literature search was conducted in PubMed (MEDLINE), Embase, Scopus, Web of Science, and the Cochrane CENTRAL Register from database inception to May 2025. The search strategy was customized for each database using a combination of controlled vocabulary (e.g., MeSH) and free-text keywords related to midshaft clavicle fractures, operative and nonoperative management. To ensure completeness, reference lists of all included studies and relevant systematic reviews were manually screened to identify additional eligible articles.

Inclusion and Exclusion Criteria

 The inclusion and exclusion criteria used for study selection are summarized in Table 1.

**Table 1:** Inclusion and exclusion criteria DASH: Disabilities of the Arm, Shoulder, and Hand score

Inclusion criteria	Exclusion criteria
Direct comparison of surgical vs non-surgical management of displaced midshaft clavicle fractures	Case reports, review articles, or conference abstracts
Adult patients (≥18 years) with closed, acute fractures (Within six weeks of the index trauma)	Cadaveric or biomechanical studies
Reported at least one clinical outcome: union rate, nonunion, malunion, Constant score, DASH score, complications, or patient satisfaction	Non-comparative studies, Studies with insufficient or incomplete outcome data
Peer-reviewed publications	Studies involving pediatric populations (<18 years)
Published in English	Open fractures

Outcome Measures

The primary outcomes assessed were fracture union, nonunion, malunion, and functional scores using the Constant Shoulder Score (CS) and the Disabilities of the Arm, Shoulder, and Hand (DASH) score. Secondary outcomes included time to radiographic union, cosmetic satisfaction, complication rates, the need for secondary procedures (e.g., implant removal), and overall patient-reported satisfaction.

Data Extraction

Reviewers independently extracted data from each study using a standardized data collection form. Extracted information included study design, sample size, patient demographics, fracture classification, treatment modality (surgical vs. non-surgical), follow-up duration, clinical and functional outcomes, and reported complications. Discrepancies between reviewers were resolved through discussion or by consultation with a third reviewer.

Quality Assessment

The methodological quality of the included studies was evaluated using the Downs and Black checklist. This tool assesses five domains: reporting quality, external validity, internal validity (bias and confounding), and statistical power. Based on total scores (maximum 28), studies were categorized as excellent (≥25), good (21-24), fair (17-20), or poor (<17).

Statistical Analysis

Meta-analyses were performed using Review Manager (RevMan) version 5.4 (The Cochrane Collaboration, London, England, UK). For continuous outcomes, such as DASH and Constant scores, standardized mean differences (SMDs) with 95% confidence intervals (CIs) were calculated. For dichotomous outcomes, including nonunion rates, odds ratios (ORs) with 95% CIs were computed.

Heterogeneity among studies was assessed using the Chi² test and quantified with the I² statistic, where I² values greater than 50% indicated moderate to high heterogeneity. A fixed-effects model was employed for analyses with low heterogeneity, while a random-effects model was used when substantial heterogeneity was detected.

Potential publication bias was evaluated through visual inspection of funnel plots and statistically using Egger’s regression test.

Results

Search and Study Selection

A systematic literature search was conducted across PubMed, Scopus, Web of Science, and Google Scholar to identify studies comparing operative versus non-operative treatment for displaced midshaft clavicle fractures. The search included all literature published up to May 2025, using terms such as “clavicle fracture,” “midshaft clavicle,” “displaced clavicle,” “operative treatment,” “non-operative treatment,” “conservative management,” “open reduction internal fixation,” “ORIF,” “DASH score,” and “Constant score.”

The initial search yielded 172 records, of which 31 duplicates were removed, leaving 141 articles for screening by title and abstract. Following this, 89 studies were excluded for not meeting eligibility criteria, which required studies to: (1) directly compare operative and non-operative management of displaced midshaft clavicle fractures; (2) report at least one relevant clinical outcome (e.g., union rate, malunion, nonunion, Constant score, DASH score, complications, or patient satisfaction); and (3) include adult patients with closed, acute midshaft clavicle fractures.

**Figure 1:**
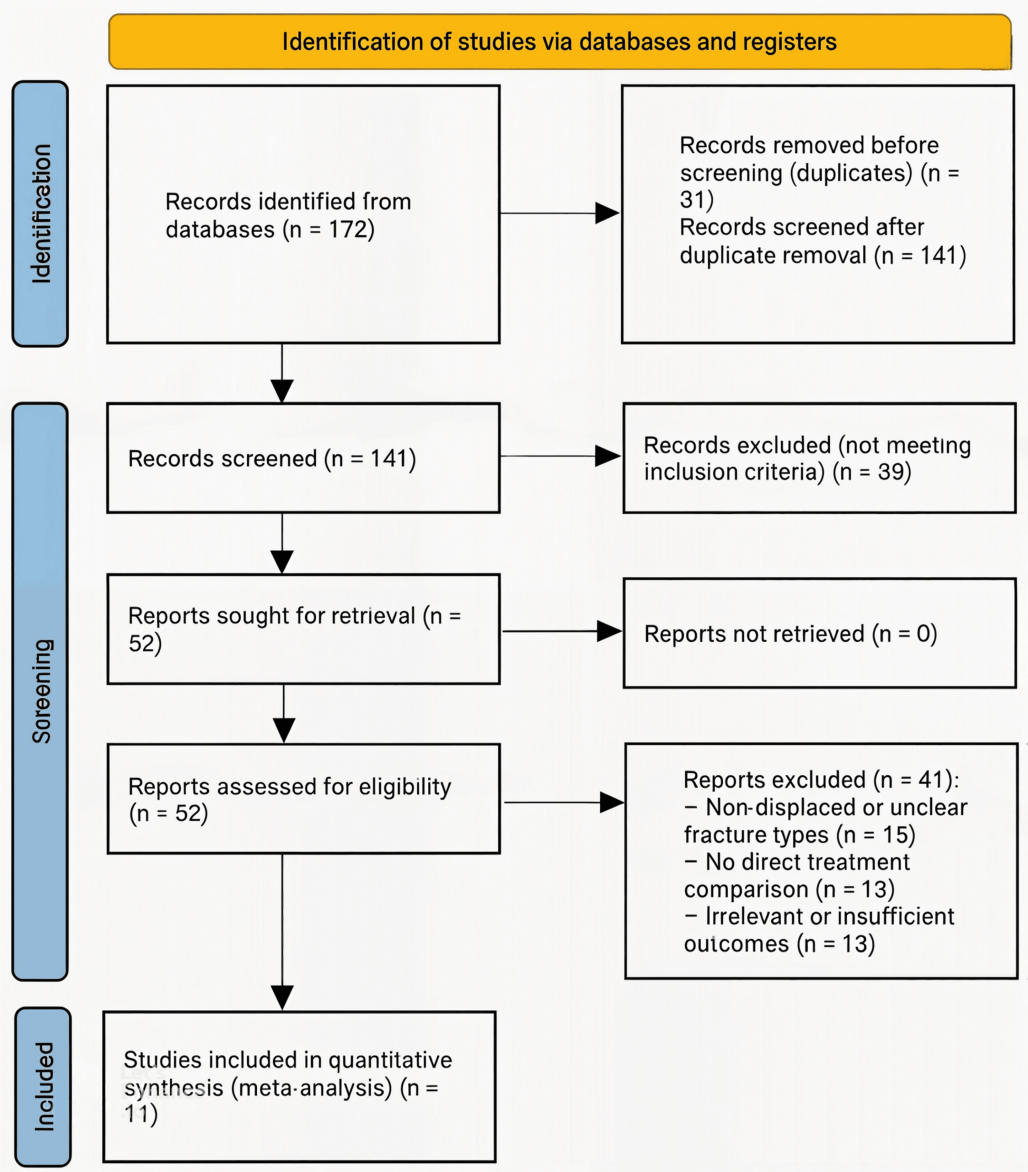
PRISMA flow diagram detailing the study selection process for the included studies comparing operative versus non-operative management of displaced midshaft clavicle fractures ORIF: open reduction and internal fixation; DASH: Disabilities of the Arm, Shoulder and Hand score; PRISMA: Preferred Reporting Items for Systematic Reviews and Meta-Analyses

Fifty-two full-text articles were then assessed for eligibility. Forty-one were excluded for the following reasons: non-comparative study design (defined as studies lacking a direct comparison between operative and non-operative management) (n = 13); inclusion of pediatric populations (n = X); insufficient outcome data (defined as studies not reporting union rates, functional scores such as Constant-Murley or DASH, or complication rates) (n = 13); non-displaced or unclear fracture patterns (n = 15); or high risk of bias. Ultimately, 11 studies met all inclusion criteria and were included in the qualitative synthesis and meta-analysis. The study selection process is detailed in the PRISMA (Preferred Reporting Items for Systematic Reviews and Meta-Analyses) flow diagram (Figure 1).

Study Characteristics

This meta-analysis includes eleven studies comparing operative versus non-operative treatment for displaced midshaft clavicle fractures, encompassing a total of 1,084 patients treated across diverse clinical settings and geographic regions. The included studies varied in design and evidence level, consisting of randomized controlled trials (RCTs), prospective comparative cohorts, retrospective analyses, and non-randomized trials, with evidence levels ranging from I to III.

Participants were predominantly adults aged 16 to 65 years, with a majority being young to middle-aged males, consistent with the demographic typically affected by high-energy trauma or road traffic accidents. All studies focused on displaced midshaft clavicle fractures, mainly classified as Robinson types 2B1 and 2B2. The operative interventions involved open reduction and internal fixation (ORIF), primarily using pre-contoured or dynamic compression plates. Non-operative treatments commonly included immobilization with figure-of-eight bandages or slings.

Follow-up periods ranged from three months to over two years, with most studies assessing outcomes at six and 12 months. Primary outcome measures included the Constant score, DASH score, radiographic union rates, time to union, rates of malunion and nonunion, revision surgeries, and patient satisfaction.

Operative protocols generally involved fixation within one to two weeks post-injury, followed by standardized rehabilitation programs. Non-operative management typically entailed immobilization for three to six weeks with subsequent gradual mobilization. Criteria for surgical versus non-surgical treatment varied among studies and were often based on fracture displacement, patient activity levels, or cosmetic concerns.

Across the included studies, operative treatment consistently resulted in lower rates of nonunion and malunion, particularly in younger, more active patients and those with significantly displaced fractures. Although long-term functional outcomes at final follow-up were often comparable between groups, surgery was frequently associated with faster functional recovery, earlier return to work, and greater cosmetic satisfaction. However, surgical intervention also carries risks, including implant irritation and the need for hardware removal.

A summary of the included studies, detailing sample sizes, patient demographics, intervention details, complications, follow-up durations, and key outcomes, is provided in Table 2.

**Table 2:** Summary of study characteristics, including sample size, patient demographics, interventions, follow-up duration, complications, and primary outcomes for studies comparing operative versus non-operative management of displaced midshaft clavicle fractures CS: Constant score; DASH: Disabilities of the Arm, Shoulder and Hand score; ROM: range of motion; ORIF: open reduction and internal fixation; DCP: dynamic compression plate; RCT: randomized controlled trial

Study	Study design	Sample size	Level of evidence	Patient demographics	Intervention details	Follow-up duration	Outcome measures	Results	Complications	Conclusions
Ranjan et al. [12]	Prospective randomized study	60 patients (30 operative, 30 non-operative)	Level II	Mean age: ~36.7 years; 86.7% male; side: 75% left clavicle; Robinson 2B1 and 2B2 fractures included	Operative: Plate fixation using superior surface LCP or recon plate within 7 days. Non-operative: Clavicle brace (rucksack bandage) for 6 weeks. Same rehab in both groups. Evaluated at 2, 4, 6 wks; 3, 6, and 12 months.	12 months	CS score, DASH score, fracture union, malunion, shortening, complications	CS and DASH scores significantly better in operative group at 3 months (CS p=0.0469, DASH p=0.0406). No significant difference at 1 year. Union: 100% operative vs. 80% non-op. Nonunion in 5 non-op cases (20%). Malunion in 2, shortening in 3, muscle wasting in 2 in non-op group. Satisfaction with appearance: 100% operative vs. 60% non-op (p=0.041).	Non-op: 23 complications (NU 20%, malunion 8%, shortening 12%, pain 32%). Operative: 4 complications (2 implant-related, 1 implant failure, 1 irritation).	Operative management offers faster functional recovery, lower nonunion and malunion rates, and better cosmetic outcomes. Non-operative treatment associated with more complications and delayed healing. Surgical fixation is a strong option in active adults with displaced midshaft clavicle fractures.
Shetty et al. [25]	Prospective comparative study	30 patients (16 operative, 14 non-op)	Level II	Adults aged 20–50 years; 25 males, 5 females; AO type A/B midshaft clavicle fractures with mild-moderate displacement	Operative group: Open reduction and internal fixation (locking compression plate). Non-operative group: Clavicle brace and arm pouch immobilization for 3 weeks. DASH scores recorded at 3, 6, and 24 weeks. Both groups began shoulder rehab after 3 weeks.	24 weeks	DASH score (shoulder function), radiographic union, mal-union, non-union	Final DASH at 24 weeks: Operative group 7.75 ± 16.42, Non-op group 8.57 ± 6.07 (NS). At 6 weeks: Operative group 53.38, Non-op 59.07 (p = 0.254). No non-unions in either group. Mal-union occurred in 6/14 patients in the non-op group; none in the operative group.	6 mal-unions in non-op group; none in operative group; no non-unions in either group	Operative fixation reduces mal-union in midshaft clavicle fractures but shows no significant difference in shoulder function at 24 weeks compared to conservative management. Functional outcomes are comparable, though surgical treatment avoids deformity-related complications.
Woltz et al. [26]	Multicenter RCT (Sleutel Trial)	160 patients (86 operative, 74 non-op)	Level I	Adults aged 18–60 with Robinson type 2B1/2B2 fractures; majority male (~91%); similar age/smoking/trauma types in both groups	Operative group: ORIF with plate fixation within 3 weeks; Non-op group: sling + rehab. Outcomes measured at 6 weeks, 3 months, and 12 months. Standardized rehab protocols across groups. Radiographs assessed union; Constant and DASH for function; SF-36 for general health.	1 year	Nonunion, Constant & DASH scores, secondary operations, pain, cosmetic satisfaction, general health (SF-36)	Nonunion: 2.4% (operative) vs. 23.1% (non-op) (p<0.0001); Secondary fixation for nonunion: 1.2% vs. 12.9% (p=0.006); No significant difference in Constant/DASH scores at any time point; Cosmetic dissatisfaction: 5% (operative) vs. 18% (non-op, p=0.06); Pain scores and SF-36 physical slightly better at 6 wks for operative group but no difference long-term; Functional outcomes similar at 12 months.	Secondary ops: 27.4% (operative, incl. 16.7% elective implant removal) vs. 17.1% (non-op); deep infections (2), early/late implant failure (6), neurologic (1)	Plate fixation significantly reduces nonunion in displaced midshaft clavicle fractures but does not improve long-term functional outcomes. High reoperation rate in both groups. Routine surgery not recommended for all; non-op is reasonable for most unless early high-demand use or strong patient preference for surgery.
Tamaoki et al. [27]	Multicenter RCT	98 completed (47 nonsurgical, 51 surgical)	Level I	Mean age: nonsurgical 34.6 yrs, surgical 30.5 yrs (p=0.046); similar sex, trauma mechanism, dominance, fracture type	Surgical group: Anterior plate fixation (3.5 mm reconstruction plate). Nonsurgical group: figure-of-eight harness. Both groups followed the same rehab protocol. Primary outcome: DASH at 6 months. Secondary: VAS pain, time to return to work, cosmetic satisfaction, radiographic healing, complications.	1 year	DASH, VAS pain, radiographic healing, nonunion, shortening, cosmetic satisfaction, return to work	DASH scores and VAS pain were not significantly different at any point. Nonunion: 0% in surgical group vs. 14.9% in nonsurgical (p=0.004). Clavicle shortening significantly greater in nonsurgical (0.93 cm vs. 0.48 cm, p<0.001). More cosmetic dissatisfaction, malpositioning, and bone prominence in nonsurgical group. Paresthesia: 13.7% surgical vs. 2.1% nonsurgical (p=0.036). Return to work slightly earlier in surgical group (NS). No ROM restriction in either group.	7 nonunions in nonsurgical (2 required surgery); paresthesia in 7 surgical vs 1 nonsurgical; 3 hardware removals in surgical	No functional benefit of surgery over nonsurgical treatment using DASH scores, but surgery significantly reduced nonunion and anatomical deformities. Choice of treatment should consider patient preference, risk of nonunion, and cosmetic expectations. Surgical treatment showed more nerve-related symptoms; both approaches are viable with distinct risk profiles.
Micheloni et al. [28]	Retrospective comparative cohort study	87 patients (50 operative, 37 non-op)	Level III	Surgical group: 45M/5F, mean age 36.8; Non-op group: 28M/9F, mean age 46.8; AO type 15.2A fractures	Operative: ORIF with plate fixation within 2 weeks. Non-operative: Figure-of-eight bandage. Constant and DASH scores assessed. Complications recorded including malunion, cosmetic dissatisfaction, and reoperation.	Mean 48 months	Constant score, DASH score, complication rate, cosmetic satisfaction	Constant score: 94.36 (operative) vs. 91.56 (non-op). DASH: 4.63 vs. 3.86 (non-op better). No significant difference (p > 0.5). Non-union and malunion more frequent in non-op group. Aesthetic dissatisfaction: 39.5% (non-op) vs. 12% (op). Implant removal in 20% of surgical group; 13.9% of non-op patients needed delayed surgery for malunion.	Non-op: 39.5% aesthetic dissatisfaction, 13.9% surgical conversion for malunion. Op: 20% implant removal, 14% sensory issues, 1 wound dehiscence	Plate fixation reduces non-union and malunion but does not significantly improve functional outcomes. Cosmetic and structural deformities more common in conservative group. Surgical option preferred for young, active patients with higher union demands. Treatment should be individualized.
Lenza et al. [29]	Prospective comparative study	102 patients (52 conservative, 50 surgical)	Level II	Conservative: mean age 36 yrs; Surgical: mean age 42 yrs; RTA most common cause (~85%); both genders included	Surgical: Open reduction and internal fixation using clavicular “S” plate ± lag screw. Conservative: figure-of-eight bandage. All patients followed ≥1-year. DASH and Constant scores assessed. Union defined radiologically; functional ROM evaluated.	≥1 year	DASH score, Constant score, time to union, complication rate	DASH: 7.9 ± 2.1 (conservative) vs 10.8 ± 1.1 (surgical); Constant: 90.2 ± 15 vs 93.1 ± 13; Union time: 12.3 wks vs 9.7 wks (NS). Complication rate: 40.3% (conservative) vs 28% (surgical), p=0.001. Conservative group had higher malunion (19.2%), cosmetic dissatisfaction (11.5%), 15 required surgical correction. Operative group had 3 nonunions (6%), 3 plate breakages (6%), 5 screw backouts (10%).	Conservative: 21/52 complications (malunion, esthetic dissatisfaction, surgical conversion). Surgical: 14/50 complications (hardware-related, nonunion).	Both groups had similar clinical and radiological outcomes at 1 year. However, the surgical group had a significantly lower complication rate. Conservative treatment had more malunions and esthetic issues requiring surgical correction. Operative management is preferred when aiming to minimize complications in displaced midshaft clavicle fractures.
Naveen et al. [30]	Prospective non-randomized comparative trial	60 patients (30 non-op, 30 operative)	Level II	Adults aged 20–50; majority male; Robinson type 2B1 and 2B2 midshaft clavicle fractures	Non-operative: Figure-of-eight bandage + sling. Operative: ORIF with superior 3.5mm DCP plate within 7 days. Standardized rehab; Constant scores measured at 6 wks, 3 mo, 6 mo. Radiographic and clinical union monitored.	6 months	Constant score, time to union, subjective satisfaction, union rate, complications	Mean Constant scores significantly better in surgical group at all timepoints (p<0.001). Time to union shorter: 9.3 wks (op) vs. 11.3 wks (non-op). Union: 100% op vs. 93% non-op. Satisfaction higher in op (83% vs. 73%). Malunion: 6 (non-op) vs. 1 (op). Nonunion: 2 (non-op) vs. 0. No difference in overall complication rate (p=0.371).	Conservative: 30% complications (malunion, nonunion, ROM restriction). Operative: 20% (scar, hardware prominence).	Operative fixation in displaced midshaft clavicle fractures leads to faster union, better Constant scores, and fewer malunions/nonunions. Conservative treatment remains standard for non-displaced fractures, but surgery is preferred for displaced or comminuted fractures in active adults. Both options are viable depending on fracture type and patient activity level.
Melean et al. [31]	RCT in worker’s compensation population	76 patients (42 conservative, 34 surgical)	Level I	Adults (mean age ~37); all had Robinson 2B1 or 2B2 fractures; all covered by workers’ compensation	Surgical: ORIF with 3.5 mm locking compression plate or reconstruction plate. Conservative: sling for 3 weeks, then physiotherapy. CT scans assessed healing at 6 and 12 weeks. Constant score measured at 3, 6, and 12 months. Primary outcome: time to complete return to work. Secondary: bone union, functional outcomes, complications.	12 ± 2 months	Time to return to work, CT union at 6/12 weeks, Constant score, nonunion, revision surgery	Return to work: 2.9 ± 0.8 mo (surgical) vs 3.7 ± 1.1 mo (conservative), p=.003. CT healing at 6 wks: 24.1% (surgical) vs 5.3% (conservative), p=.05. At 12 wks: 81% vs 16.7%, p=.005. Constant score at 6 months: 85 (surgical) vs 81 (conservative), p=.004; at 12 months: 93 vs 87, p=.003. Nonunion: 0 (surgical) vs 4 (conservative); all 4 needed surgery. 4 surgical patients had hardware removal.	Conservative: 4 nonunions (9.6%). Surgical: 4 implant removals (11.7%) due to discomfort. No infections or neurovascular issues.	ORIF led to earlier return to work, faster radiographic union, and fewer nonunions than conservative treatment. Constant scores were better in the surgical group at 6 and 12 months. For laborers with displaced midshaft clavicle fractures, surgical fixation offers a clear advantage in functional and socioeconomic recovery.
Jha et al. [32]	Prospective clinical trial	60 patients (30 conservative, 30 operative)	Level II	Mean age: 32.2 years; 78.3% male; 51.7% left side affected; Robinson Type 2B1/2B2	Conservative: Figure-of-eight bandage and arm sling. Operative: ORIF with 3.5 mm plate. All followed same rehab protocol. Union, function, and satisfaction assessed at each follow-up.	6 months	Time to union, Constant score, malunion, nonunion, patient satisfaction	Union: 16.04 wks (conservative), 14.57 wks (operative), p=0.191. Constant score: 94.47 (cons) vs. 96 (op), p=0.445. Malunion: 70% (cons) vs. 3.3% (op), p<0.001. Satisfaction: 70% (cons) vs. 93.3% (op), p=0.02.	Conservative: 2 nonunions, 21 malunions. Operative: 1 infection, 4 screw loosening, 2 implant failures	Both groups achieved similar union time and functional scores. However, the conservative group had significantly more malunions and lower patient satisfaction. Operative fixation is recommended for improved alignment and satisfaction in displaced fractures.
Ahrens et al. [33]	Multicenter RCT	301 patients (154 surgical, 147 non-op)	Level I	Adults aged 18–65 years with displaced midshaft clavicle fractures (Robinson 2B1 or 2B2); balanced by age/gender	Surgical: ORIF using pre-contoured titanium plate (Acumed system); standardized rehab protocol. Non-op: arm sling, early ROM after 2 weeks, active rehab after 6 weeks. Outcomes assessed at 6 weeks, 3 months, and 9 months.	9 months	Primary: Non-union at 3 months. Secondary: DASH, Constant scores, patient satisfaction	3-month union rate: No difference (28% nonunion in surgical vs. 27% in non-op; p=0.87). At 9 months: 11% nonunion in non-op vs. 0.8% in op (p<0.001). DASH and Constant scores significantly better in op group at 6 weeks and 3 months; no difference at 9 months. Early satisfaction higher in op group. 11% of non-op group required secondary surgery. Functional recovery slower in non-op group.	Operative: 1 re-op for loss of fixation, no surgical site infections. Non-op: 11% nonunion; 11% required surgery later.	Operative fixation leads to lower nonunion rate at 9 months, better early function and satisfaction, but both groups had similar long-term outcomes. ORIF is a safe and reliable option, especially when early return to function or lower nonunion risk is desired. Conservative treatment carries a higher secondary surgery rate and should be selected based on patient preference and functional needs.
Mughal et al. [34]	Cross-sectional comparative study	150 patients (75 operative, 75 conservative)	Level III	Operative and conservative groups: each had 59 males (78.7%) and 16 females (21.3%); age 16–60 yrs; mean 33.65 yrs	Operative: ORIF with small dynamic compression plate (DCP). Conservative: polysling immobilization. Nonunion assessed at 4–6 months post-treatment clinically and radiographically.	4–6 months	Non-union rate	Nonunion: 5 (6.66%) in operative vs. 14 (18.66%) in conservative group (p=0.047). Higher nonunion especially in patients over 40 yrs in conservative group.	Conservative: 18.6% nonunion. Operative: 6.7% nonunion, minor hardware-related issues (e.g., infection, prominence).	Surgical fixation significantly reduced nonunion rates compared to conservative treatment, especially in younger patients. Operative fixation is recommended for displaced midshaft fractures to ensure anatomical alignment and reduce complications. Conservative management had higher rates of delayed healing and nonunion.

Functional outcomes were primarily assessed using the Constant score [35] and the DASH questionnaire [36], both of which are freely available for clinical and academic research and do not require licensing or special permissions.

Quality Assessment of Included Studies

The methodological quality of the eleven included studies was assessed using the Downs and Black checklist, a validated tool designed to evaluate both randomized and non-randomized studies across five key domains: reporting, external validity, internal validity (bias and confounding), and statistical power [37]. Each study received a score out of a maximum of 28 points, with higher scores indicating greater methodological rigor [37]; meta-analyses were subsequently conducted using RevMan 5.4 (The Cochrane Collaboration, London, UK) [38].

Overall, the studies demonstrated moderate to high quality. They performed particularly well in the domains of reporting and internal validity related to bias. However, there was greater variability in external validity and adjustment for confounding factors, especially among retrospective cohort and non-randomized comparative studies. Randomization and blinding procedures were generally well-documented in the randomized controlled trials, whereas observational studies showed more limited control for potential confounders.

Of the included studies, three were rated as Excellent (scores ≥25), five as Good (scores 21-24), and three as Fair (scores 17-20). No studies were classified as Poor (<17), underscoring the overall robustness of the evidence base included in this meta-analysis.

Detailed Downs and Black scores for each study, broken down by domain and total score, along with the overall quality rating, are summarized in Table 3.

**Table 3:** Quality assessment of included studies using the Downs and Black checklist Scores reflect methodological performance across five key domains with ratings categorized as: Excellent (≥25), Good (21–24), Fair (17–20), and Poor (<17).

Study (citation)	Study design	Reporting (0–11)	External validity (0–3)	Internal validity – bias (0–7)	Internal validity – confounding (0–6)	Power (0–1)	Total score (max 28)	Quality rating
Ranjan et al. [12]	Prospective randomized trial	9	2	6	5	1	23	Good
Shetty et al. [25]	Prospective comparative study	8	2	5	4	0	19	Fair
Woltz et al. [26]	Multicenter RCT	10	3	7	6	1	27	Excellent
Tamaoki et al. [27]	Multicenter RCT	9	3	6	6	1	25	Good
Micheloni et al. [28]	Retrospective cohort	7	2	5	4	0	18	Fair
Lenza et al. [29]	Prospective comparative study	9	3	6	5	1	24	Good
Naveen et al. [30]	Prospective non-randomized trial	8	2	6	5	1	22	Good
Melean et al. [31]	RCT in worker population	10	3	7	6	1	27	Excellent
Jha et al. [32]	Prospective clinical trial	8	2	5	4	0	19	Fair
Ahrens et al. [33]	Multicenter RCT	10	3	7	6	1	27	Excellent
Mughal et al. [34]	Cross-sectional comparative study	7	2	5	4	0	18	Fair

Results of Meta-Analysis

Disability (DASH score): The meta-analysis assessing functional disability via the DASH score revealed no statistically significant difference between surgical and non-surgical treatment groups (Standardized Mean Difference (SMD) = 0.27; 95% Confidence Interval (CI): -0.30 to 0.84; p = 0.35). Although the pooled estimate showed a slight trend favoring surgery, the confidence interval crossed zero, indicating the difference was not statistically significant.

There was substantial heterogeneity among the included studies (Chi² = 45.28, degrees of freedom (df) = 5, p < 0.00001; I² = 89%), which likely reflects variations in follow-up durations, fracture types, rehabilitation protocols, and timing of DASH score assessments.

**Figure 2:**
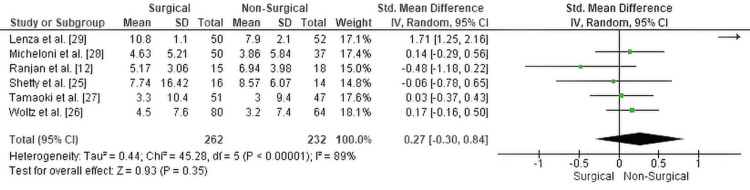
Forest plot comparing DASH scores between surgical and non-surgical management of displaced midshaft clavicle fractures SMD: standardized mean difference; CI: confidence interval; DASH: Disabilities of the Arm, Shoulder and Hand score

The corresponding forest plot illustrating these results is presented in Figure 2.

Publication bias assessment for DASH score:** **The funnel plot for the DASH score demonstrates a relatively symmetrical distribution of studies around the vertical line, with no significant outliers and most points clustered near the pooled effect estimate. This visual symmetry suggests an absence of publication bias for this outcome. Although one study is positioned further to the right, the overall distribution does not indicate asymmetry.

Additionally, Egger’s regression test was non-significant (p > 0.05), further supporting the lack of small-study effects or publication bias.

**Figure 3:**
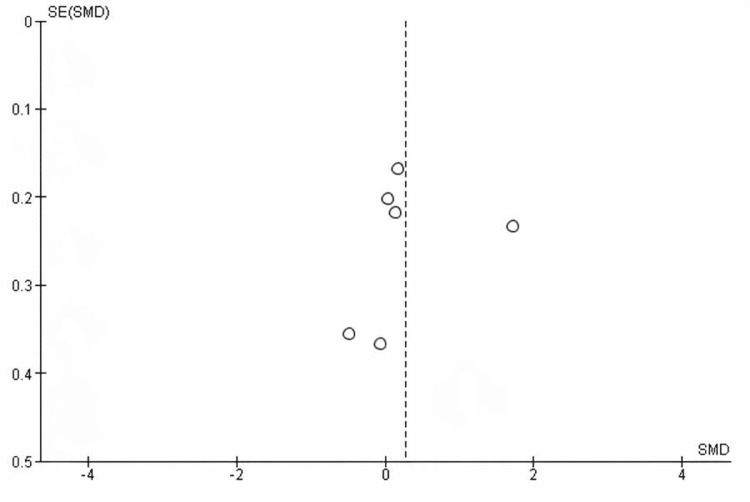
Funnel plot assessing publication bias for DASH scores in studies comparing surgical versus non-surgical treatment of displaced midshaft clavicle fractures Funnel plot of standardized mean differences (SMD) versus standard error (SE) for Disabilities of the Arm, Shoulder and Hand (DASH), showing no evidence of publication bias (Egger’s test p > 0.05).

The funnel plot for this analysis is shown in Figure 3.

Shoulder function (Constant score): The meta-analysis assessing shoulder function using the Constant Shoulder Score demonstrated a statistically significant advantage for surgical treatment (SMD = 0.49, 95% CI: 0.01 to 0.98, p = 0.05). This finding suggests that patients who underwent operative fixation experienced moderately better shoulder function compared to those managed non-operatively.

However, there was substantial heterogeneity among the included studies (Chi² = 45.27, df = 6, p < 0.00001; I² = 87%), indicating variability that may be attributed to differences in follow-up durations, surgical techniques, or patient demographics.

**Figure 4:**
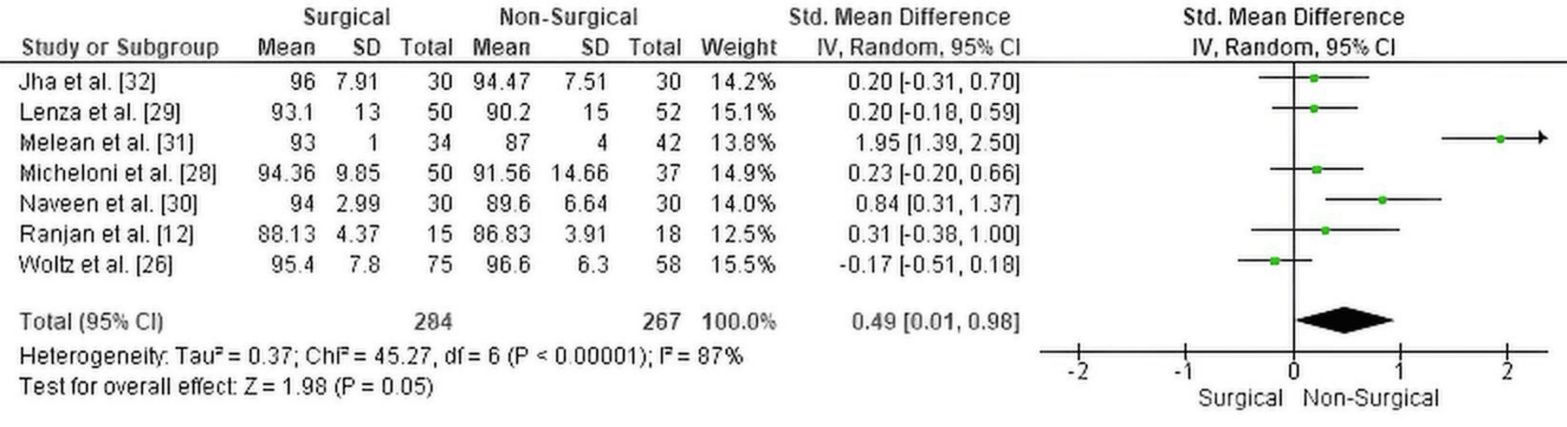
Forest plot comparing Constant Shoulder Scores between surgical and non-surgical management of displaced midshaft clavicle fractures SMD: standardized mean difference; CI: confidence interval

The forest plot for this analysis is presented in Figure 4.

Publication bias assessment for Constant Shoulder Score: The funnel plot for the Constant Shoulder Score reveals a slightly asymmetrical distribution, with a few studies positioned to the right of the mean effect, which may suggest a mild publication bias or small-study effects. Nevertheless, most studies remain reasonably clustered without any extreme outliers.

Given the limited number of included studies (n = 7), the power of visual assessment is constrained. Additionally, Egger’s regression test did not reach statistical significance (p > 0.05), indicating no strong evidence of publication bias for this outcome.

**Figure 5:**
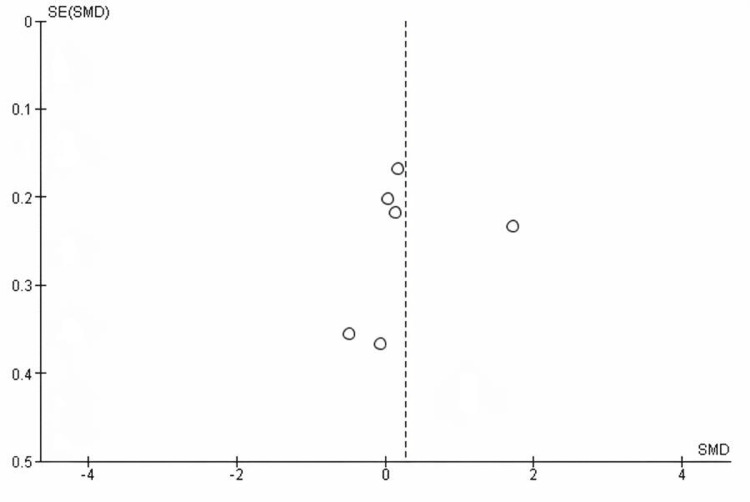
Funnel plot assessing publication bias for Constant Shoulder Scores in studies comparing surgical versus non-surgical treatment of displaced midshaft clavicle fractures SMD: standardized mean difference, SE: standard error

Figure 5 displays the funnel plot for this analysis.

Nonunion: The meta-analysis assessing nonunion rates demonstrated a statistically significant advantage for surgical treatment over non-surgical management (OR = 0.23, 95% CI: 0.12 to 0.42, p < 0.00001). This result indicates that patients undergoing surgical fixation had a 77% lower likelihood of developing nonunion compared to those treated conservatively. The analysis showed low heterogeneity among studies (Chi² = 8.48, df = 8, p = 0.39; I² = 6%), reflecting consistent findings across the included trials.

**Figure 6:**
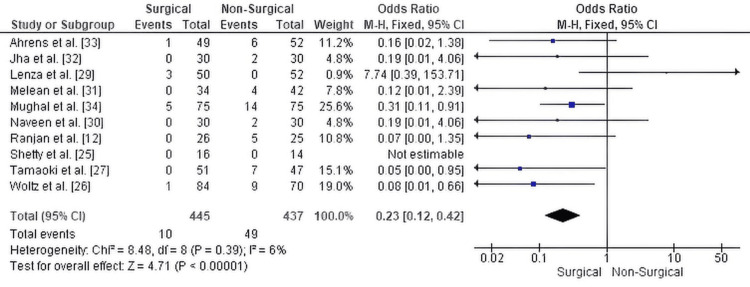
Forest plot comparing nonunion rates between surgical and non-surgical management of displaced midshaft clavicle fractures OR: odds ratio; CI: confidence interval.

Figure 6 illustrates the forest plot summarizing these results.

**Figure 7:**
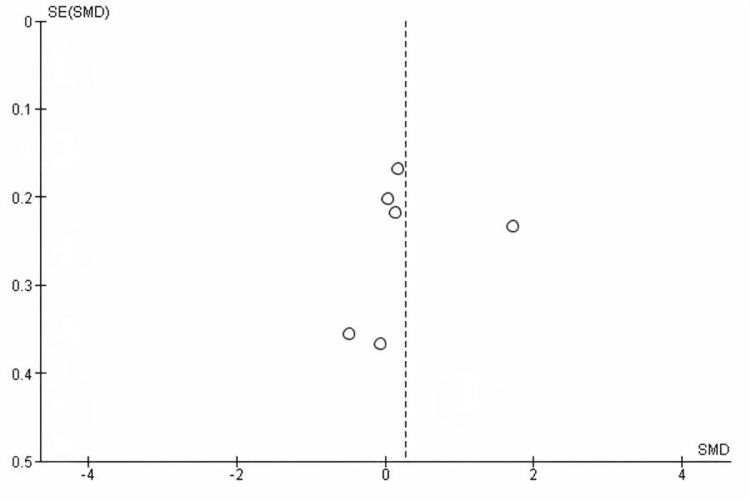
Funnel plot assessing publication bias for nonunion rates in studies comparing surgical versus non-surgical management of displaced midshaft clavicle fractures. OR: odds ratio, SE: standard error

Publication bias assessment for nonunion: The funnel plot assessing publication bias for nonunion rates displays some asymmetry, with one study appearing as an outlier on the far right, indicating a higher odds ratio compared to the others. Most studies cluster to the left of the mean effect, which may suggest the presence of small-study effects or potential publication bias. However, given the relatively small number of included studies, this visual asymmetry should be interpreted cautiously. Egger’s regression test was not statistically significant (p > 0.05), indicating no strong statistical evidence of publication bias despite some visual concerns. Figure 7 shows the funnel plot for this analysis.

Discussion

This systematic review and meta-analysis evaluated the comparative effectiveness of surgical versus non-surgical management in adult patients with displaced midshaft clavicle fractures. Eleven studies comprising 1,084 patients across diverse clinical settings and study designs-including randomized controlled trials (RCTs), prospective, and retrospective cohort analyses-were included. The review focused on important clinical outcomes such as union rates, functional scores, complications, patient satisfaction, and cosmetic results.

The findings indicate that surgical treatment confers significant benefits in reducing rates of nonunion and malunion, improving early functional outcomes, and enhancing cosmetic satisfaction, particularly in active adults and those with markedly displaced fractures. The pooled data demonstrated a notably lower risk of nonunion in the operative group (OR = 0.23, p < 0.00001), consistent with major RCTs such as those by Woltz et al. and Ahrens et al.**,** which reported nonunion rates as high as 23% in non-operative groups compared to less than 3% in surgically treated patients [26,33]. This consistency across high-quality studies reinforces the reliability of the observed treatment effect.

Surgical management was associated with improved Constant Shoulder Scores (SMD = 0.49, p = 0.05), indicating better shoulder strength and range of motion. However, the DASH scores-reflecting broader upper limb disability-showed no statistically significant difference between groups (SMD = 0.27, p = 0.35). This suggests that while surgical patients may experience earlier or more complete recovery of shoulder function, the perceived disability in everyday activities may equalize over time. These findings are supported by studies such as Tamaoki et al. and Micheloni et al., where early functional advantages of surgery diminished at longer follow-up [27,28].

Cosmetic outcomes and patient satisfaction also tended to favor surgical treatment. Several studies, including Ranjan et al. and Jha et al., reported higher satisfaction in operative cohorts, largely due to improved anatomical alignment and reduced deformity [12,32]. Malunion and shortening-common in conservative treatment-adversely affected both cosmetic satisfaction and perceived function, especially in fractures with vertical displacement or shortening greater than 2 cm. This highlights the importance of personalized management, as patients with specific fracture characteristics or cosmetic concerns may benefit most from operative fixation.

Nonetheless, surgical intervention is not without risks. Although nonunion and malunion rates were lower in the operative group, complications such as implant irritation, hardware prominence, screw loosening, infection, and the need for secondary surgeries like hardware removal were reported. For example, Woltz et al. documented a reoperation rate of 27.4%, including 16.7% elective implant removals [26]. These potential iatrogenic complications necessitate careful risk-benefit assessment, especially in patients with lower functional demands or comorbidities contraindicating surgery.

Long-term functional outcomes (beyond 12 months) appeared comparable between surgical and non-surgical groups, suggesting non-operative treatment remains a reasonable option in selected patients. This was illustrated in studies like Micheloni et al. and Lenza et al.**,** where final Constant and DASH scores were similar despite earlier recovery differences [28,29]. This aligns with the emerging consensus that surgical fixation accelerates rehabilitation and early function but may not significantly improve long-term results.

The substantial heterogeneity in meta-analyses of DASH (I² = 89%) and Constant scores (I² = 87%) likely reflects variability in study protocols, patient demographics, fracture classifications, and follow-up durations. While sensitivity analyses were limited due to the number of studies, the consistent effect directions and use of random-effects models support the robustness of conclusions. The low heterogeneity observed in the nonunion analysis (I² = 6%) strengthens the evidence favoring surgical intervention to reduce nonunion risk.

An evolving area warranting attention is the influence of socioeconomic and healthcare system factors on treatment decisions. Surgical management tends to be more frequently offered in high-resource environments and to patients with private insurance or treated in adult hospitals [22]. In that study, the authors reported that insurance status and hospital type were independent predictors of operative intervention, with privately insured patients and those treated in adult hospitals being more likely to undergo surgical fixation. This highlights potential access disparities that warrant further investigation from a health equity perspective.

Ultimately, treatment decisions for displaced midshaft clavicle fractures should be individualized, integrating clinical factors such as fracture displacement, comminution, patient age, activity level, and cosmetic concerns. Shared decision-making informed by objective criteria (e.g., displacement >100%, shortening >2 cm), predictive tools, and the evidence presented here may optimize patient outcomes and satisfaction.

Limitations

This review has several limitations. First, variability in study designs, follow-up durations, and rehabilitation protocols contributed to heterogeneity across the included studies. Second, the inclusion of non-randomized studies increased the risk of selection bias. Third, inconsistent reporting of cosmetic outcomes and complications limited the ability to perform comprehensive comparisons. Finally, although formal tests did not detect significant publication bias, the possibility cannot be completely excluded due to the relatively small number of studies.

## Conclusions

Surgical fixation of displaced midshaft clavicle fractures offers clear advantages, including reduced risk of nonunion and malunion, faster early recovery, and improved short-term functional and cosmetic outcomes compared to non-surgical management. Despite these early benefits, long-term functional outcomes are generally comparable between operative and conservative approaches. Treatment decisions should therefore be individualized, considering fracture severity, patient activity level, cosmetic expectations, and personal preference. Both operative and non-operative strategies remain valid, though plate fixation is particularly beneficial for young, active patients with significantly displaced fractures.

## References

[1] Bhatia S, Greenspoon JA, Petri M, Millett PJ (2017). Midshaft clavicle fracture: open reduction and internal fixation. Case Competencies in Orthopaedic Surgery.

[2] Raju GB, Ravish VN, Gowda BM (2024). Surgical treatment of midshaft clavicle fractures with intramedullary titanium elastic nailing system. J Orthop Dis Traumatol.

[3] Hoffmeister E (2008). Recent research in treating midshaft clavicle fractures. Lippincott’s Bone Joint Newsl.

[4] Denard PJ, Koval KJ, Cantu RV, Weinstein JN (2005). Management of midshaft clavicle fractures in adults. Am J Orthop (Belle Mead NJ).

[5] Vivekanandan R (2013). Functional Outcome of Midshaft Clavicle Fracture Treated with Titanium Elastic Nail System: Short Term Prospective Outcome Analysis (Thesis). https://core.ac.uk/download/pdf/235658146.pdf.

[6] Nicholson JA, Clement ND, Clelland AD, MacDonald D, Simpson AH, Robinson CM (2020). Displaced midshaft clavicle fracture union can be accurately predicted with a delayed assessment at 6 weeks following injury: a prospective cohort study. J Bone Joint Surg Am.

[7] Ranalletta M (2020). CORR Insights®: What is the best evidence for management of displaced midshaft clavicle fractures? A systematic review and network meta-analysis of 22 randomized controlled trials. Clin Orthop Relat Res.

[8] Rüst CA, Knechtle B, Knechtle P, Rosemann T (2011). Atrophy of the brachialis muscle after a displaced clavicle fracture in an Ironman triathlete: case report. J Brachial Plex Peripher Nerve Inj.

[9] Hoogervorst P, Konings P, Hannink G, Holla M, Schreurs W, Verdonschot N, van Kampen A (2020). Functional outcomes, union rate, and complications of the Anser Clavicle Pin at 1 year: a novel intramedullary device in managing midshaft clavicle fractures. JSES Int.

[10] Campbell DH, McKee MD (2020). Operative fixation of a displaced midshaft clavicle fracture. J Orthop Trauma.

[11] Kulshrestha V, Roy T, Audige L (2011). Operative versus nonoperative management of displaced midshaft clavicle fractures: a prospective cohort study. J Orthop Trauma.

[12] Ranjan N, Agarwal A, Garg A (2019). A comparative study of conservative and surgical management of displaced midshaft clavicle fracture. Truama Int.

[13] Pawar E, Pohokar A, Kambli M, Modi N, Bansal S, Mishra S (2021). Conservative vs operative management of displaced midshaft clavicle fracture: a comparative study. Int J Case Rep Orthop.

[14] Cha SD, Chung ST, Kim YH, Park SJ (2010). The analysis of conservative treatment in midshaft fractures of clavicle. Clin Shoulder Elbow.

[15] van der Ven Denise JC, Timmers TK, Flikweert PE, Van Ijseldijk AL, van Olden GD (2015). Plate fixation versus conservative treatment of displaced midshaft clavicle fractures: Functional outcome and patients' satisfaction during a mean follow-up of 5 years. Injury.

[16] Lee GB, Kim H, Jeon IH, Koh KH (2021). Long-term outcomes of initially conservatively treated midshaft clavicle fractures. Clin Shoulder Elb.

[17] Randsborg PH, Fuglesang HF, Røtterud JH, Hammer OL, Sivertsen EA (2014). Long-term patient-reported outcome after fractures of the clavicle in patients aged 10 to 18 years. J Pediatr Orthop.

[18] Tutuhatunewa ED, Stevens M (2017). Clinical outcomes and predictors of patient satisfaction in displaced midshaft clavicle fractures in adults: results from a retrospective multicentre study. Injury.

[19] Schneider P, Bransford R, Harvey E, Agel J (2019). Operative treatment of displaced midshaft clavicle fractures: has randomised control trial evidence changed practice patterns?. BMJ Open.

[20] Subramanyam KN, Mundargi AV, Gopakumar KU, Bharath T, Prabhu MV, Khanchandani P (2021). Displaced midshaft clavicle fractures in adults - is non-operative management enough?. Injury.

[21] Axelrod DE, Ekhtiari S, Bozzo A, Bhandari M, Johal H (2020). What is the best evidence for management of displaced midshaft clavicle fractures? A systematic review and network meta-analysis of 22 randomized controlled trials. Clin Orthop Relat Res.

[22] Zhang D, Heyworth BE, Liotta ES, Hergott KA, Earp BE (2021). Variation in treatment approaches to adolescent midshaft clavicle fractures in pediatric versus adult hospitals. J Orthop Trauma.

[23] Jørgensen A, Troelsen A, Ban I (2014). Predictors associated with nonunion and symptomatic malunion following non-operative treatment of displaced midshaft clavicle fractures--a systematic review of the literature. Int Orthop.

[24] Gao B, Dwivedi S, Patel SA, Nwizu C, Cruz AI Jr (2019). Operative versus nonoperative management of displaced midshaft clavicle fractures in pediatric and adolescent patients: a systematic review and meta-analysis. J Orthop Trauma.

[25] Shetty SK, Chandran R, Ballal A, Mathias LJ, Hegde A, Shetty A (2017). To operate or not to operate the mid-shaft fractures of the clavicle: a comparative study of functional outcomes of the two methods of management. J Clin Diagn Res.

[26] Woltz S, Stegeman SA, Krijnen P (2017). Plate fixation compared with nonoperative treatment for displaced midshaft clavicular fractures: a multicenter randomized controlled trial. J Bone Joint Surg Am.

[27] Tamaoki MJ, Matsunaga FT, Costa AR, Netto NA, Matsumoto MH, Belloti JC (2017). Treatment of displaced midshaft clavicle fractures: figure-of-eight harness versus anterior plate osteosynthesis: a randomized controlled trial. J Bone Joint Surg Am.

[28] Micheloni GM, Tarallo L, Porcellini G, Catani F (2019). Comparison between conservative treatment and plate fixation for displaced middle third clavicle fracture: clinical outcomes and complications. Acta Biomed.

[29] Lenza M, Buchbinder R, Johnston RV, Ferrari BA, Faloppa F (2019). Surgical versus conservative interventions for treating fractures of the middle third of the clavicle. Cochrane Database Syst Rev.

[30] Naveen BM, Joshi GR, Harikrishnan B (2017). Management of mid-shaft clavicular fractures: comparison between non-operative treatment and plate fixation in 60 patients. Strategies Trauma Limb Reconstr.

[31] Melean PA, Zuniga A, Marsalli M, Fritis NA, Cook ER, Zilleruelo M, Alvarez C (2015). Surgical treatment of displaced middle-third clavicular fractures: a prospective, randomized trial in a working compensation population. J Shoulder Elbow Surg.

[32] Jha GK, Timsina P, Yadav D, Lamichhane S, Jha S (2018). Conservative vs operative management of displaced midshaft clavicle fracture: a comparative study. Biomed J Sci Tech Res.

[33] Ahrens PM, Garlick NI, Barber J, Tims EM (2017). The clavicle trial: a multicenter randomized controlled trial comparing operative with nonoperative treatment of displaced midshaft clavicle fractures. J Bone Joint Surg Am.

[34] Mughal AH, Khan MS, Javed M, Amanullah A (2016). Comparison of outcome of conservative versus operative management of displaced midshaft clavicle fractures. Gomal J Med Sci.

[35] Constant CR, Murley AG (1987). A clinical method of functional assessment of the shoulder. Clin Orthop Relat Res.

[36] Hudak PL, Amadio PC, Bombardier C (1996). Development of an upper extremity outcome measure: the DASH (disabilities of the arm, shoulder and hand). Am J Ind Med.

[37] Downs SH, Black N (1998). The feasibility of creating a checklist for the assessment of the methodological quality both of randomised and non-randomised studies of health care interventions. J Epidemiol Community Health.

[38] (2020). Review Manager (RevMan), Version 5.4. https://test-training.cochrane.org/online-learning/core-software-cochrane-reviews/review-manager-revman/download-revman-5.

